# Our Contemporaries

**Published:** 1917-07

**Authors:** 


### OUR CONTEMPORARIES

The March issue of the Polyclinic of London consists, with a single exception, of abstracts from medical journals of the U. S. The exception is from a South African source.

La Revue de Chimiothérapie has recently begun issue from Paris, with Dr. J. Laumorier as editor.

We acknowledge the courtesy of the Journal of Sociologic Medicine, published by the American Academy of Medicine, the "University Club" of the medical profession, in quoting at length, our editorial on Health Insurance, published in March.

